# Dynamic Modeling and Flow Distribution of Complex Micron Scale Pipe Network

**DOI:** 10.3390/mi12070763

**Published:** 2021-06-28

**Authors:** Yao Zhao, Kai Zhang, Fengbei Guo, Mingyue Yang

**Affiliations:** College of Metrology & Measurement Engineering, China Jiliang University, Hangzhou 310018, China; s1802080449@cjlu.edu.cn (Y.Z.); s20020804019@cjlu.edu.cn (F.G.); s1902080445@cjlu.edu.cn (M.Y.)

**Keywords:** microfluidic, flow distributions, fluid network

## Abstract

A fluid simulation calculation method of the microfluidic network is proposed as a means to achieve the flow distribution of the microfluidic network. This paper quantitatively analyzes the influence of flow distribution in microfluidic devices impacted by pressure variation in the pressure source and channel length. The flow distribution in microfluidic devices with three types of channel lengths under three different pressure conditions is studied and shows that the results obtained by the simulation calculation method on the basis of the fluid network are close to those given by the calculation method of the conventional electrical method. The simulation calculation method on the basis of the fluid network studied in this paper has computational reliability and can respond to the influence of microfluidic network length changes to the fluid system, which plays an active role in Lab-on-a-chip design and microchannel flow prediction.

## 1. Introduction

Nowadays, the rapid development of science and technology constantly drives the upgrading of scientific and technological products. The portability, integration and intelligentization of scientific and technological products are the key points and difficulties in current science and technology product development. Meanwhile, with the improvement of living standards, health issues have become the focus of people’s attention so that people put forward higher standards for the accuracy of test results and detection methods. However, the real-time detection and rapid diagnosis technology in medical and health fields are currently less developed and need to be improved urgently. Therefore, the microfluidic chip technology arises at the right moment.

Microfluidic chip technology refers to building biological or chemical laboratories on chips that are only a few square centimeters in size. Different types of fluid channels, whose diameters are in the dozens to hundreds of micrometers range, are built on the microfluidic chip based on various functions. These channels have many utilities, such as biological or chemical reactions and extraction or detection of reagents. Meanwhile, the microfluidic transmission process can be controlled through the design of the microchannel network [1].

The main functions of the microfluidic system are transferring and mixing, reaction separation, and flow control of microfluidics in the microchannels. Because of the small size of the fluid channel, the fluid flowing in the microchannel is mostly in the laminar flow state, such as particles, droplets or bubbles, which generally belong to the field of low Reynolds number fluid theory in microchannels [2].

In order to calculate and control the flow of the microfluidic network more accurately, the study method that is commonly used by researchers is the electrical equivalent method [3]. By using the electrical equivalent method to liken the micropipe to an electrical device, the microfluidic network can be integrated and analyzed. However, it cannot simulate the issues of the local resistance well in the microfluidic pipe, and the diverting resistance in the microfluidic network needs to be calculated by mathematic methods to be more accurate [4]. This manner aforementioned is the calculation method of a fluid network which is on the basis of the three conservation equations of basic fluid mechanics, including the conversation of mass, momentum and energy [5]. Pressure value and flow rate can be obtained by simultaneous calculations of mass and momentum and heat is solved by the energy equation. However, this study method is rarely reported at present.

Recently, the flow characteristics of microfluidic networks have been studied by many scholars. However, most of them focus on computational fluid dynamics (CFD) and electrical analogy methods [3,6]. For example, Ali Y. Alharbi’s group studied the flow process of fractal-like branching networks in micropipes and the pressure drop characteristics and force mode in branching microchannels using three-dimensional computational fluid dynamics approaches [7]. Dalei Jing and Yongping Chen et al. also studied the heat and mass transfer in branched microfluidic channels, but the equivalent methods of microfluidic resistance are all hypotheses of using circuits [8,9]. Bassiouny’s group [10,11] developed analytical equations to predict the fluid flow distribution in rectangular, U-shaped and Z-shaped manifolds. Kim’s group [12] analyzed the influence of a Z-manifold on channel flow distribution and studied three different shapes, including rectangle, trapezoid and triangle. Delsman’s group [6] performed a numerical analysis of the flow distribution in a multi-channel microfluidic device with Z-shape and inline manifolds. They obtained the results of multiple manifold designs by changing the shape and flow direction of the device’s inlet and outlet. Wang’s group [13] took friction and momentum effects into account in the manifold and modified the U-shaped manifold model established by Bassiouny and Martine; they found that the effects of friction and momentum could increase and decrease the pressure drop in the manifold, respectively. Therefore, it is observed that a uniform distribution of fluid flow can be achieved by balancing these two opposite effects properly.

In this paper, we use the fluid network calculation method to analyze the flow characteristics of the microfluidic network and the conservation equation of mass and momentum to solve the pressure and flow in the microfluidic network. The method that we used was on the basis of the structural characteristics and flow process of the actual network. The flow distribution in a microfluidic device with five microchannels is analyzed and compared with the conventional electrical method. The fluid network method takes the formula of resistance term in the calculation program into account, and its calculation results are closer to the actual flow process than those using the conventional electrical method.

## 2. Model Development

In this study, it is assumed that the fluid flowing in the microfluidic network is water and that there is no phase change during transportation. Therefore, it can be considered a single-phase incompressible fluid. The hypotheses made to establish the model in this study can be expressed as follows:

(a) No power source in the fluid network model.
(b) The fluid state in the nodes is uniform (the internal pressure is equal).
(c) The flow resistance only takes the equivalent frictional resistance along the pipeline into account and keeps the flow resistance coefficient constant.
(d) The cross-sectional area in the same branch pipe remains unchanged, and the working medium parameters are represented by the weighted average of the connected node parameters.

The construction of the microfluidic pipe network model mainly includes the following two parts:

(a) Nodes, including pipe transitions and other essential components in the microfluidic network.
(b) Branches, a connecting component between two nodes.

**Figure 1:**
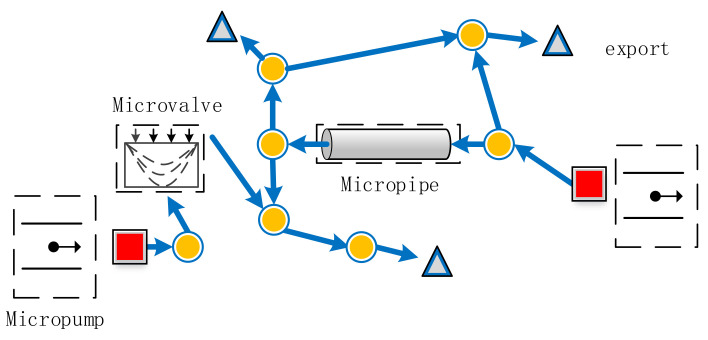
Directed topology model of the microfluidic network.

The directed topology model of the microfluidic network is shown in Figure 1. In the microfluidic network, both the micropump, as a power source, and the network outlet are regarded as boundary nodes. Moreover, the intermediate branches include various resistance components, such as a micropipe and microvalve.

For a stable microfluidic network, the continuity equation (continuous mass) in the flow process can be expressed as follows:

(1) $$V_{i}\frac{d\rho_{i}}{dt}=\sum_{j=1}^{N}D_{ij}G_{ij}$$

Without considering the influence of heat from $\frac{d\rho }{dt}=\frac{\partial \rho }{\partial p}\frac{dp}{dt}+\frac{\partial \rho }{\partial H}\frac{dH}{dt}\approx \frac{\partial \rho }{\partial p}\frac{dp}{dt}$, the following equation can be obtained:

(2) $$C_{i}\frac{dp_{i}}{dt}=\sum_{j=1}^{N}D_{ij}G_{ij}$$

where, $C_{i}=V_{i}\frac{\partial \rho_{i}}{\partial p_{i}}$ is the compressibility (kg/MPa) of the working fluid; p is the pressure of the working fluid; ρ is the density of the working fluid; t is the time; *N* is the total number of nodes; $G_{ij}$ is the mass flow between nodes *i* and *j*; $V_{i}$ is the volume of node *i*; $D_{ij}$ is the connection mode between nodes *i* and *j* $\left(i=1,2,\ldots ,N;j=1,2,\ldots ,N\right)$ and the specific meaning is:

$$D_{ij}\left\{\begin{array}{c} 1,\mathrm{there}\;\mathrm{is}\;a\;\mathrm{connection}\;\mathrm{between}\;\mathrm{nodes}\;i\;\mathrm{and}\;j,\;\mathrm{and}\;\mathrm{the}\;\mathrm{flow}\;\mathrm{direction}\;\mathrm{is}\;\mathrm{from}\;j\;\mathrm{to}\;i\\ 0,\mathrm{there}\;\mathrm{is}\;\mathrm{no}\;\mathrm{connection}\;\mathrm{between}\;\mathrm{nodes}\;i\;\mathrm{and}\;j\\ -1,\mathrm{there}\;\mathrm{is}\;a\;\mathrm{connection}\;\mathrm{between}\;\mathrm{nodes}\;i\;\mathrm{and}\;j,\;\mathrm{and}\;\mathrm{the}\;\mathrm{flow}\;\mathrm{direction}\;\mathrm{is}\;\mathrm{from}\;i\;\mathrm{to}\;j \end{array}\right.$$

**Figure 2:**
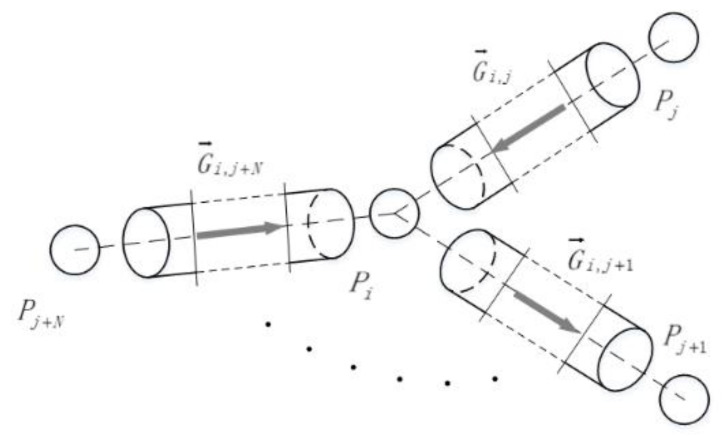
Schematic diagram of the topological structure of the *i*-th node of the pipeline. *P* is the pressure at node *i*. $G_{ij}$ is the flow rate between node *i* and node *j*.

The continuity equation is the flow conservation relationship at a node, and the flow state of the node is affected by the nodes connected to it. Figure 2 shows a schematic diagram of the volume elements of all nodes which are connected to *i*. It is important to note that a node can be connected to *N* nodes logically. However, a node to connect the number of other nodes is limited in the actual model.

The momentum conservation equation in the microfluidic network can be expressed as follows:

(3) $$\rho_{ij}L_{ij}\frac{dU_{ij}}{dt}=D_{ij}\times \left(P_{j}-P_{i}+D_{ij}\times H_{ij}\right)-h_{w}$$

where, $H_{ij}$ is the pressure generated by macro kinetic energy, potential energy and power source between node *i* and *j*; $U_{ij}$ is the fluid velocity between node *i* and *j*; $D_{ij}$ has the same meaning as $D_{ij}$ in Equations (1) and (2).

In the formula, $h_{w}=\sum \left[0.5\lambda \frac{L}{d}+\xi \right]$ is the on-way and local resistance loss [14]; λ is the on-way resistance coefficient; *L* is the length of pipe; *d* is the diameter of the pipe; *ξ* is the local pressure loss.

According to the pipeline inertia coefficient $I_{ij}=\frac{L_{ij}}{A_{ij}}$, the friction resistance coefficient on the pipeline $R_{ij}=\frac{h_{w}}{{\left(A_{ij}u_{ij}\rho_{ij}\right)}^{2}}$, and no power supply in the pipeline, $H_{ij}=0$ is introduced into the Equation (3):

(4) $$I_{ij}\frac{dG_{ij}}{dt}=D_{ij}\times \left(P_{j}-P_{i}\right)-R_{ij}\times G_{ij}^{2}$$

$R_{ij\;}$ is the resistance characteristic term between node *i* and *j*, which can be expanded as follows:

(5) $$R_{f}=\left(\frac{\lambda L}{d}+\xi \right)\frac{1}{2\rho A^{2}}$$

where, λ is the equivalent resistance coefficient on the way of the microchannel; ξ is the local resistance coefficient of the microchannel; ρ is the working medium density; A is the cross-sectional area of the microchannel.

**Figure 3:**
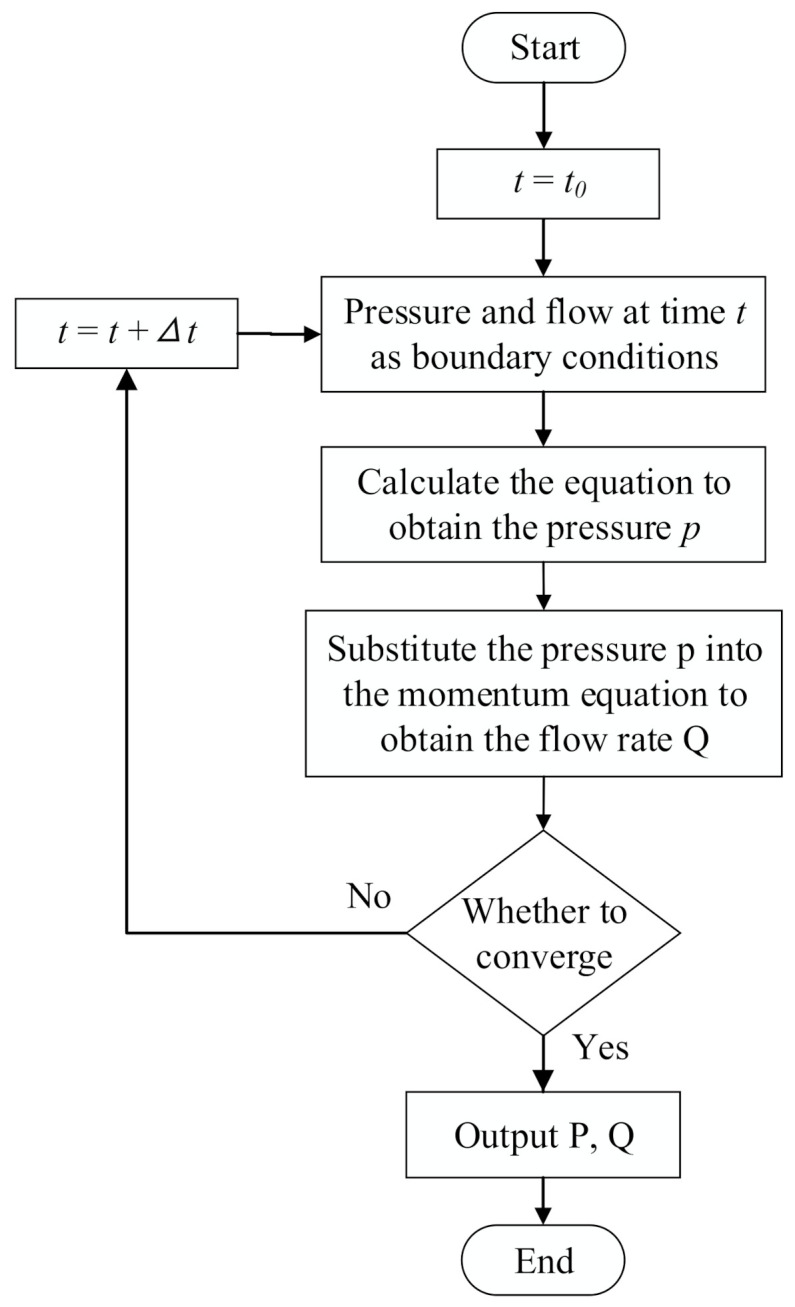
Schematic diagram of calculation process using fluid network method.

Equations (2) and (4) are nonlinear differential and algebraic equations. For dynamic simulation, the priority is to ensure real-time performance, which requires high robustness of the calculation process. Using an implicit Euler integration algorithm to calculate, the equation can be obtained by equations (2) and (4) as follows:

(6) $$C_{i}^{r+1}\frac{P_{i}^{r+1}-p_{i}^{r}}{\Delta t}=\sum_{j=1}^{N}D_{ij}^{r}\sqrt{\frac{D_{ij}^{r}\left(p_{j}^{r+1}-p_{i}^{r+1}\right)}{R_{ij}}}$$

where, *r* is the discrete-time variable, time is $t=t_{0}+r\Delta t,r=0,1,2,\ldots$ Equation (6) is completely decoupled. The pressure of each node can be obtained by solving the *N*-order nonlinear algebraic equations composed by equation (6) in the process of dynamic simulation. Under the slight perturbation condition, $C_{i}^{t+1}$ can be substituted by $C_{i}^{t}$, which has little influence on precision and can reduce the calculation amount. After solving the pressure of each node, the momentum equation is introduced to obtain the branch flow. The calculation process is shown in Figure 3.

## 3. Results and Discussions

### 3.1. Model Design and Electrical Equivalent

The basic structure of the microfluidic system model includes micropump, micromixer, microvalve, and microchannel with appropriate size. The flow characteristics of the fluid in the chip can be analyzed at the micron or even nanometer level through these unique miniaturized structures and control devices.

**Figure 4:**
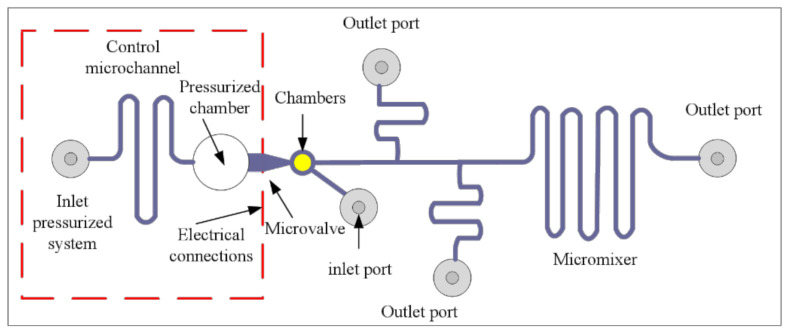
Structure of the microfluidic platform.

In this case, taking the microscopic scale issues of fluid flow into account, a fluid flow model of an incompressible fluid with a low Reynolds number is adopted to study pipeline pressure distribution under microscale conditions and flow distribution characteristics in the process of fluid flow. Therefore, we designed a microfluidic flow distribution model that has a single entry with three exports, such as Figure 4, as a simulation tool. The variation of fluid flow value in different pipe segments under different inlet pressures was observed and compared with the results obtained by using the electrical method. Since there is no mixing of the two fluids, in this case, the micromixer is not considered in the design of the structure, and the position of the microvalve does not affect the final equivalence study of the fluid flow distribution. Therefore, the electrical analogy method can treat microchannels as resistors.

Figure 4 shows the specific device design of the microfluidic model. The first is the drive system, which consists of a pressurized chamber, a microchannel controller, and a microvalve that activates the pulse. According to the actual characteristics of our pipe network, we choose the pressure pump as the driving device, whose main role is to provide driving pressure for the microfluidic network. The size of the pressure chamber can be determined on the basis of the size of the microvalve and microchannel used. In the electrical model, this is a power supply that provides voltage. The pressurized chamber is one of the ways to provide the driving pressure. Through the combination of the pressure chamber and the micro valve as a controllable pressure source, the driving pressure can be adjusted according to the system demand.

**Figure 5:**
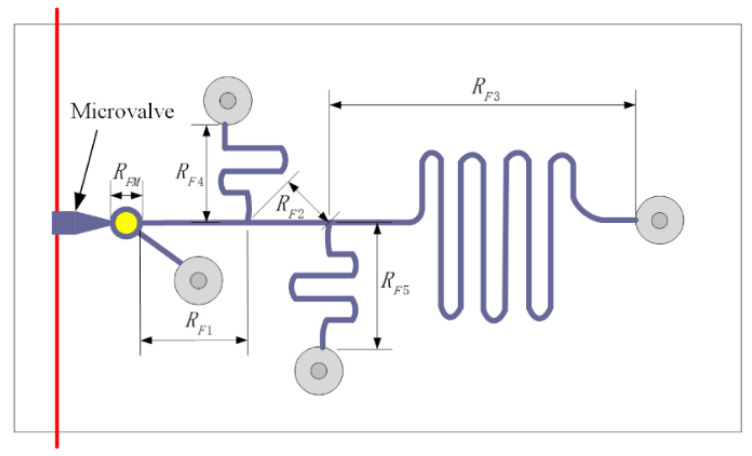
Fluidic resistors of the microfluidic circuit.

**Figure 6:**
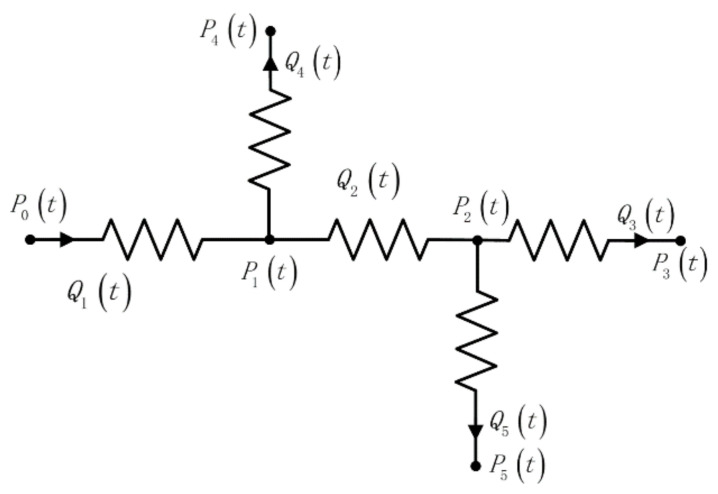
Electrical model of microfluidic platform.

In order to verify the accuracy of our algorithm, we have mentioned previously the conventional electrical equivalence method in which the microfluidic network is equivalent to a circuit model and the fluid flow parameters are equivalent to electrical parameters. According to the electrical abstraction of our model, Figure 4, the microfluidic network with circuit characteristics can be obtained, as shown in Figure 5. Where, $R_{F1}$ is the microchannel resistance of the microvalve output, $R_{F2}$ is the main channel resistance of fluid flow, and $R_{F3}$, $R_{F4}$, $R_{F5}$ is the branch channel resistance of fluid flow, respectively. Corresponding to the resistance of the storage tank where the fluid is located and taking the processing technology of the microfluidic chip into account, the cross-section of the microfluidic tube is generally rectangular, and its resistance is [15]:

(7) $$R_{f}=\frac{12uL}{(1-0.63(h/w))h^{3}w}$$

where, *L* is the length of the microchannel, *h* is its height, *w* is its width, and *u* is the viscosity of the fluid. Because of the greater sizes of reservoir and outlet, their resistances to remaining resistors are ignored. Excluding the influence of structure and considering only the logical model of the circuit, the equivalent circuit can be obtained, as shown in Figure 6.

$P_{0}\left(t\right)$ represents the pressure in the supercharging chamber, that is, namely the pressure value at the inlet of the channel;$\;P_{1}\left(t\right)$ and $P_{2}\left(t\right)$ corresponding to the pressure value at the node of the channel; $P_{3}\left(t\right)$, $P_{4}\left(t\right)$, and $P_{5}\left(t\right)$ corresponding to the pressure value at the outlet of the channel and atmospheric pressure is considered. $Q_{1}\left(t\right)\;$ and $Q_{2}\left(t\right)$ are the fluid flows in the main channel, and $Q_{3}\left(t\right)$, $Q_{4}\left(t\right)$, and $Q_{5}\left(t\right)$ are the fluid flows in each branching channel.

In this paper, we use Simulink to build the model because of using the circuit analogy method to analyze the parameters of the fluid network in the microchannel. Simulink is a graphical modeling tool in MATLAB software, which can build and calculate various circuit models. The circuit analogy built by Simulink satisfies basic electrical laws, such as Ehrhoff’s and Ohm’s law. According to the structure in Figure 6, the relevant power parameter relationship can be obtained, and the specific expression is as follows:

(8) $$P_{0}\left(t\right)-P_{1}\left(t\right)=Q_{1}\left(t\right)R_{F1}$$

(9) $$P_{1}\left(t\right)-P_{2}\left(t\right)=Q_{2}\left(t\right)R_{F2}$$

(10) $$P_{1}\left(t\right)-P_{4}\left(t\right)=Q_{4}\left(t\right)R_{F4}$$

(11) $$P_{2}\left(t\right)-P_{3}\left(t\right)=Q_{3}\left(t\right)R_{F3}$$

(12) $$P_{2}\left(t\right)-P_{5}\left(t\right)=Q_{5}\left(t\right)R_{F5}$$

(13) $$Q_{1}\left(t\right)=Q_{3}\left(t\right)+Q_{4}\left(t\right)+Q_{5}\left(t\right)$$

(14) $$Q_{2}\left(t\right)=Q_{3}\left(t\right)+Q_{5}\left(t\right)$$

(15) $$P_{0}\left(t\right)=\frac{P_{i}V_{i}}{\left(V\left(t\right)+V_{i}\right)}$$

(16) $$V\left(t\right)=\int q\left(t\right)dt$$

### 3.2. Model Calculation Results

According to the electrical method, the equivalent resistance and the dimensions of each branching channel in this example are shown in Table 1. The main local drag coefficient in this model is brought about by the micro three-way pipe. For the time being, we do not consider the local resistance coefficient ξ, but only consider the resistance of the micropipe, which is compared with the circuit model of the electrical method.

In order to reduce the activation energy required for fluid flow, the microvalve needs to have a higher length-width ratio. A PDMS film deformation valve can be selected, and both the height of the valve and the main channel are designed to be 500 μm, whose main role is to control the inlet pressure of the fluid network. The driving pressure here is the network pressure, so the micro-valve resistance does not need to be considered. Meanwhile, considering the uniformity of the fluid flow in the microchannel, the distance of the fluid movement should be as long as possible, and the cross-sectional area design of the fluid flow channel should be narrow. Therefore, the length percentages of channels 3, 4, and 5 are set to 5:3:3 and the height is set to 350 μm.

Above all, we keep other parameters constant and change the driving pressure, $P_{0}\left(t\right)$, in the microfluidic network, set as 15 kPa, 18 kPa, and 20 kPa, respectively. In the outlet boundary condition of the microchannel, the pressure values, $P_{3}\left(t\right)$, $P_{4}\left(t\right)$ and $P_{5}\left(t\right)$, are set as 11 kPa, 12 kPa and 11 kPa, respectively. The results of the fluid network algorithm and electrical simulation of Simulink are shown in Table 2. Both the results indicate that the flow in the microfluidic network satisfies the relationship of flow distribution. The flow rate of $Q_{2}$ in the main microchannel is the sum of $Q_{3}$ and $Q_{5}.$ The flow rate of $Q_{1}\;$ is the sum of $Q_{2}$ and $Q_{4}$. Moreover, there is a certain nonlinear relationship among the microchannels when the input pressure of the pressure pump increases. The electrical method is very similar to the results of the fluid network method we used, which proves the correctness of the fluid network method and can be used to calculate the microfluidic network.

**Figure 7:**
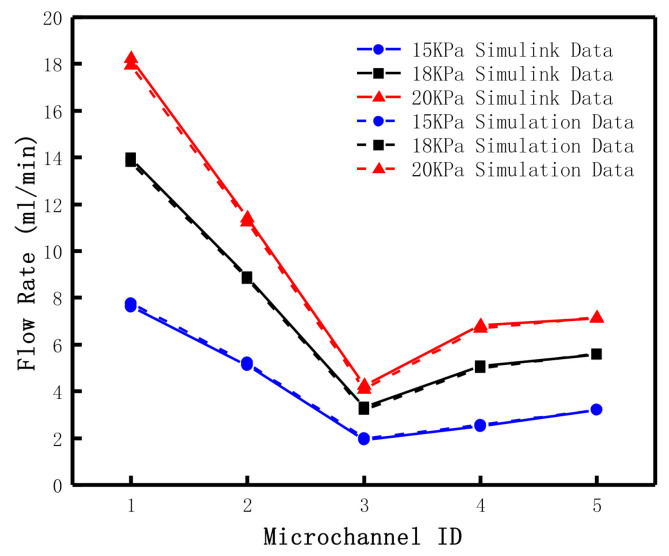
Schematic diagram of different driving pressures.

The first example shows that the total flow rate of the system,$\;Q_{1}\left(t\right),$ increases with the increase of the input pressure. Microchannel 3 is still the outlet with a smaller flow rate, due to the longer length of microchannel 3, so its equivalent resistance value is greater than that of microchannel 4 and 5. Afterward, the increase of pressure leads to the flow rate rise, which is mainly reflected in the shunting of micro-channel 4 and 5. While the flow rate increase in micro-channel 3 is relatively lower. Both calculation results show this point, as shown in Figure 7, and can prove that the computational results of the fluid network method are closer to those of the electrical method, which has certain computational accuracy.

**Figure 8:**
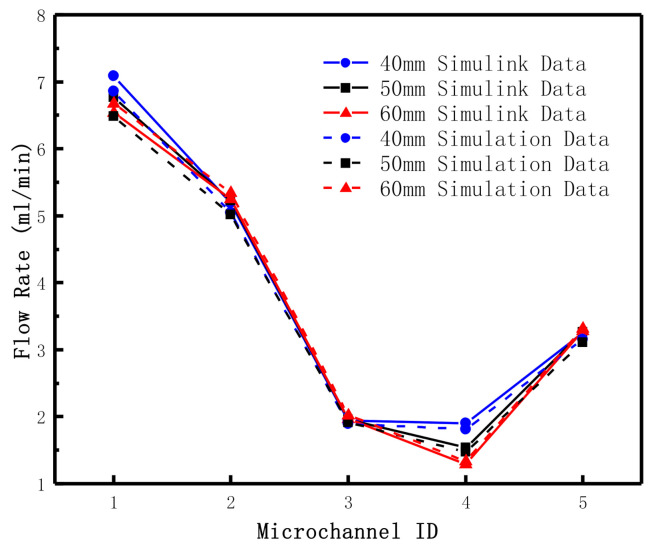
Schematic diagram of calculation results under different lengths of microchannel 4.

In the second example, in order to study the influence of microchannel structure on flow characteristics, the width and depth of microchannel 4 were kept at 350 μm. The flow distributions at different channel lengths (40 mm, 50 mm and 60 mm) were studied. The boundary condition at the inlet was set as 15 kPa and the three outlet pressures,$\;P_{3}\left(t\right)$, $P_{4}\left(t\right)$, and $P_{5}\left(t\right)$, are set as 11 kPa, 12 kPa, and 11 kPa, respectively. The calculation results of the two methods are shown in Table 3 and Figure 8. The diagram shows that the flow rate in microchannel 4 decreases with the increase of its length, which is because the increase of the length of the microchannel leads to the resistance value rise. On the other hand, comparing microchannel 3 with microchannel 5, it can be found that the flow rate in these two channels is relatively stable. Under certain pressure conditions, the flow rate change caused by changing the structure of microchannel 4 does not affect the flow rate in microchannel 3 and 5.

In the first example in this section, we have verified the calculation accuracy of the fluid network algorithm and electrical methods. Compared the results, which are similar. However, the resistance items of the fluid network algorithm can be more detailed, such as three or more pipe diversion coefficients of local resistance, and is a good way to add items in resistance,$\;R_{ij}$, in the model. Electrical methods can treat micro-channels or micro-valves as resistance items, but it is difficult to deal with local resistance items such as three-way or multi-way, and some approximate formulas are generally adopted to calculate it [15]. According to Figure 7 and Figure 8, we know that the feedback results of the fluid network method and electrical method to the pressure and structure changes in the microfluidic network are similar, which can be used for the simulation of the fluid flow process in the microfluid network and set up test-rig in the follow-up work. According to the actual measurement parameters, the calculation model can be modified to improve its accuracy.

## 4. Conclusions

In this paper, a computational method of microfluidic pipeline network on the basis of fluid network is studied and compared with the conventional electrical equivalent method. It is found that the computational results obtained by using the fluid network method are closer to those given by using the electrical method. Therefore, we believe that the computational accuracy of the fluid network method is the same as that of the electrical method. From the research results of the simulation examples established in this paper, it can be concluded that the fluid network method can reflect the fluid flow process under different pressures, and the effects caused by structural changes in the microfluidic network are consistent with the results obtained by the electrical equivalent method. However, the electrical method has limitations in the treatment of local resistance, which is difficult to deal with the network shunt, the gradual shrinkage or expansion of microchannels, etc. that can be solved in the algorithm of fluid network easily. Furthermore, the accuracy of its calculation can be improved by merely modifying the resistance term in the algorithm. The computational method of the microfluidic network that we proposed in this paper can be used as an alternative to the electrical method. The follow-up work is to study the resistance term of the model and how to deal with the various devices presented in microfluidic is an important research direction.

## Figures and Tables

**Table 1:** Dimensions of the device and values of some parameters of the setup.

Parameter	$R_{F1}$	$R_{F2}$	$R_{F3}$	$R_{F4}$	$R_{F5}$
Width (μm)	500	500	350	350	350
Height (μm)	500	500	350	350	350
Length (mm)	10	8	50	30	30
$\mathrm{Equivalent}\;\mathrm{resistance}\;(10^{9}Pa\cdot s/m^{3}$)	4.64	3.71	96.6	57.9	57.9

**Table 2:** Model Simulation and Simulink calculation results under different pressures.

Entrance Pressure (KPa)		15	18	20
$Q_{1}$ (mL/min)	Simulink Data	7.623	13.990	18.235
	Simulation Data	7.767	13.812	17.945
	Relative Error (%)	1.883	1.272	1.585
$Q_{2}$ (mL/min)	Simulink Data	5.126	8.895	11.407
	Simulation Data	5.217	8.802	11.246
	Relative Error (%)	1.772	1.054	1.414
$Q_{3}$ (mL/min)	Simulink Data	1.921	3.333	4.275
	Simulation Data	2.000	3.211	4.086
	Relative Error (%)	4.100	3.671	4.421
$Q_{4}$ (mL/min)	Simulink Data	2.497	5.095	6.827
	Simulation Data	2.550	5.011	6.700
	Relative Error (%)	2.111	1.653	1.871
$Q_{5}$ (mL/min)	Simulink Data	3.205	5.561	7.132
	Simulation Data	3.217	5.590	7.160
	Relative Error (%)	0.378	0.515	0.388

**Table 3:** Simulation results under different lengths of microchannel 4.

Microchannel 4 Length (mm)		40	50	60
$\mathrm{Microchannel}\;4\;\mathrm{Resistance}\;(10^{10}Pa\cdot s/m^{3}$)		7.73	9.66	11.6
$Q_{1}$ (mL/min)	Simulink Data	7.090	6.764	6.541
	Simulation Data	6.855	6.488	6.680
	Relative Error (%)	3.305	4.078	2.104
$Q_{2}$ (mL/min)	Simulink Data	5.188	5.226	5.252
	Simulation Data	5.047	5.016	5.342
	Relative Error (%)	2.733	4.014	1.721
$Q_{3}$ (mL/min)	Simulink Data	1.944	1.958	1.968
	Simulation Data	1.894	1.912	2.026
	Relative Error (%)	2.580	2.369	2.943
$Q_{4}$ (mL/min)	Simulink Data	1.903	1.538	1.290
	Simulation Data	1.81	1.472	1.337
	Relative Error (%)	4.863	4.297	3.663
$Q_{5\;}\left(\mathrm{mL}/\min\right)$	Simulink Data	3.244	3.267	3.284
	Simulation Data	3.152	3.104	3.316
	Relative Error (%)	2.825	5.000	0.989
